# “It’s Like a Marathon”: A Qualitative Study of Symptom Burden and Adaptation Among Patients with Oxaliplatin-Induced Peripheral Neuropathy During Treatment

**DOI:** 10.3390/curroncol33100620

**Published:** 2026-10-14

**Authors:** Yi Zhang, Jing Han, Wen Gong, Xiaoxia Qiu

**Affiliations:** 1 School of Nursing, Xuzhou Medical University, Xuzhou 221000, China; 15161680829@163.com; 2 Department of Nursing, Renji Hospital, Shanghai Jiao Tong University School of Medicine, Shanghai 200127, China; hanjing@renji.com (J.H.); gongwen@renji.com (W.G.)

**Keywords:** oxaliplatin, chemotherapy-induced peripheral neuropathy, qualitative research, symptom management, treatment continuation, oncology nursing

## Abstract

Oxaliplatin-induced peripheral neuropathy (OIPN) can substantially affect patients’ everyday lives, yet less is known about how patients appraise and adapt to these symptoms during ongoing treatment. This qualitative study explored how patients appraise and adapt to OIPN during ongoing treatment and how these experiences relate to considerations about treatment continuation. Fifteen patients receiving oxaliplatin-based chemotherapy at a tertiary hospital in Shanghai, China, completed face-to-face semi-structured interviews. Four interrelated themes were constructed: (1) evolving and embodied symptom experiences, (2) disruption and contraction of everyday life, (3) appraising and adapting to symptom uncertainty, and (4) balancing symptom burden and perceived treatment value. Participants described recurrent symptoms that could become increasingly persistent, disrupt everyday functioning, and create uncertainty about recovery. They developed individualized strategies to manage symptoms and differed in what they considered tolerable. Considerations about treatment continuation reflected the interaction of symptom burden, perceived manageability, and perceived treatment value. These findings highlight the importance of understanding how patients appraise and adapt to OIPN during treatment. They may help oncology nurses identify support needs and provide individualized information as treatment progresses.

## Simple Summary

Oxaliplatin-induced peripheral neuropathy can affect many aspects of everyday life during chemotherapy, yet patients may understand and respond to these symptoms in different ways. We interviewed 15 patients receiving oxaliplatin-based chemotherapy to explore their experiences. Patients described recurring symptoms that sometimes became more persistent, interfered with daily activities and mobility, and created uncertainty about whether they would recover. They developed their own ways of managing symptoms and differed in what they felt able to tolerate. Their views about continuing chemotherapy reflected the burden of neuropathy, how manageable the symptoms felt, and the importance they placed on treatment. These findings may help oncology nurses better recognize patients’ changing support needs and provide individualized information as treatment progresses.

## 1. Introduction

Oxaliplatin is widely used in chemotherapy regimens for gastrointestinal malignancies, particularly colorectal and gastric cancers [1,2]. However, oxaliplatin-induced peripheral neuropathy (OIPN) is a common and potentially dose-limiting neurotoxicity that predominantly affects peripheral sensory nerves [3]. It typically manifests as numbness, tingling, and cold-induced dysesthesia in the hands and feet [3], and can impair daily functioning and quality of life [4]. In more severe cases, OIPN may necessitate chemotherapy dose reduction, treatment delay, or discontinuation [5].

OIPN follows a distinctive temporal pattern that includes acute and chronic manifestations. Acute symptoms often occur around treatment cycles and are frequently triggered by cold exposure, whereas chronic sensory disturbances may become more persistent as cumulative oxaliplatin exposure increases [3,6]. Patients may therefore experience recurrent short-term disturbances alongside a gradually accumulating neuropathic burden, with the experience of OIPN changing over the course of treatment.

Understanding this symptom experience is clinically important because preventive and therapeutic options remain limited [5,7]. In addition, conventional clinician-based grading systems may not fully capture patient-reported symptom burden and functional impact [8]. During treatment, patients must accommodate symptoms that continue to evolve while receiving a therapy from which they anticipate anticancer benefit. Understanding OIPN in this setting therefore requires attention to both symptom severity and how patients appraise its consequences as treatment continues.

Qualitative studies of chemotherapy-induced peripheral neuropathy (CIPN) describe substantial effects on physical functioning, emotional well-being, everyday activities, and self-management [9,10,11]. Evidence focused specifically on OIPN remains more limited, although existing studies similarly report neurotoxic experiences, functional consequences, and emotional responses during treatment [12,13]. However, less is known about how patients appraise OIPN while oxaliplatin treatment is ongoing, how they adapt to changing symptoms, and how these experiences become incorporated into considerations about continuing oxaliplatin treatment.

The Stress, Appraisal and Coping model conceptualizes adaptation as an ongoing process of appraisal, coping, and reappraisal as demands and available resources change [14,15]. This perspective offers a useful lens for understanding how patients adapt to OIPN as symptoms and their consequences change during treatment. Guided by this perspective, this study aimed to explore how patients experience, appraise, and adapt to OIPN during oxaliplatin treatment and how these experiences relate to considerations about treatment continuation.

## 2. Materials and Methods

### 2.1. Study Design

We used a qualitative design and applied reflexive thematic analysis (RTA) within an interpretivist, experiential orientation to explore patients’ experiences of OIPN and how these experiences related to considerations about treatment continuation. This approach emphasizes the active role of the researcher in knowledge construction, conceptualizing themes as being generated through continuous engagement between the researcher and the data, rather than passively derived from it [16]. This study is reported in accordance with the Consolidated Criteria for Reporting Qualitative Research (COREQ) [17].

### 2.2. Setting and Participants

We used purposive maximum-variation sampling to recruit patients with gastrointestinal malignancies receiving oxaliplatin-based chemotherapy. Recruitment occurred in the oncology department and day chemotherapy center of a tertiary hospital in Shanghai, China, from June 2025 to March 2026.

The inclusion criteria were: (1) pathologically confirmed gastrointestinal malignancy; (2) current treatment with an oxaliplatin-containing chemotherapy regimen; (3) the presence of peripheral neuropathy symptoms of varying severity, assessed using the National Cancer Institute Common Terminology Criteria for Adverse Events (NCI-CTCAE), version 5.0 [18]; (4) sufficient ability to communicate and participate in an in-depth interview; and (5) voluntary participation with written informed consent. The exclusion criteria were: (1) pre-existing peripheral neuropathy caused by diabetes or other neurological disorders; (2) prior or concurrent treatment with other neurotoxic chemotherapeutic agents; and (3) severe comorbidities, such as significant cardiac, hepatic, or renal dysfunction.

Recruitment and analysis proceeded iteratively. Rather than relying on a predetermined sample size or saturation criterion, we judged sample adequacy against the study aim and the richness, diversity, and depth of the dataset [19]. Recruitment ended when the dataset was sufficiently rich and varied to support a meaningful thematic account of participants’ experiences.

### 2.3. Data Collection

The research team included nurses with academic and clinical oncology backgrounds. Y.Z., a nursing postgraduate researcher trained in qualitative methods, conducted all interviews and had no prior clinical relationship with participants. Before each interview, sociodemographic information was collected, while cancer site and stage, treatment regimen, treatment cycle, and cumulative oxaliplatin dose received up to the time of interview were obtained from the medical record; OIPN severity was graded by Y.Z. according to the NCI-CTCAE version 5.0 at the pre-interview assessment.

The Stress, Appraisal and Coping model informed the interview guide, which remained open enough for participants to introduce experiences and meanings they considered important (Table 1). Three pilot interviews were used to refine the guide and were excluded from the final analysis. Interviews took place in a dedicated consultation room in the oncology department, were conducted in Mandarin Chinese, and were audio-recorded with participants’ informed consent. Field notes captured contextual information. Interviews lasted approximately 30–60 min.

### 2.4. Data Analysis

Data were managed in NVivo 15 (Lumivero, Denver, CO, USA) and analyzed using Braun and Clarke’s recursive approach to RTA, moving iteratively between familiarization, coding, theme development, refinement, and writing.

All interviews were transcribed verbatim in Chinese within 48 h by the interviewer and cross-checked against the audio recordings by a second researcher. Data analysis was conducted using the original Chinese transcripts. During familiarization, Y.Z. and J.H. repeatedly read the transcripts and listened to the recordings to develop an overall understanding of participants’ experiences within their broader contexts. Y.Z. conducted the initial coding of all transcripts, generating and iteratively refining codes relevant to the research question. Y.Z. and J.H. then generated candidate themes by examining patterns of shared meaning across the dataset, which were further refined through reflexive discussions with the wider research team.

The analysis was primarily inductive and data-led, with the final themes subsequently interpreted through the lens of the Stress, Appraisal and Coping model. Representative quotations were translated into English and cross-checked against the original Chinese text by a second researcher to preserve the intended meanings of participants’ accounts.

### 2.5. Rigor

We supported rigor through reflexive engagement, transparent analytic procedures, and systematic documentation. Maximum-variation sampling broadened the range of experiences represented in the dataset. Y.Z. kept analytic records of key decisions, developing interpretations, and reflections on potential preconceptions. Regular reflexive discussions with J.H., W.G., and X.Q. were used to examine assumptions, consider alternative interpretations, and refine the thematic account rather than seek coding consensus. Themes were repeatedly considered against participants’ narratives and illustrated with representative quotations. Detailed participant and treatment contexts are provided so readers can judge the transferability of the findings to other oncology settings.

### 2.6. Ethical Considerations

This study was conducted in accordance with the principles of the Declaration of Helsinki and received ethical approval from the Ethics Committee of Renji Hospital, Shanghai Jiao Tong University School of Medicine (LY2025-166-B), in 2025. ChatGPT (GPT-5.6 Sol; OpenAI, San Francisco, CA, USA) was used during manuscript preparation and revision solely to improve language clarity, grammar, and readability. It was not used for study design, data collection, data analysis, interpretation of the findings, or development of the scientific conclusions.

## 3. Results

### 3.1. Participant Characteristics

Participant-level sociodemographic and treatment characteristics are presented in Table 2.

### 3.2. Themes

**Figure 1:**
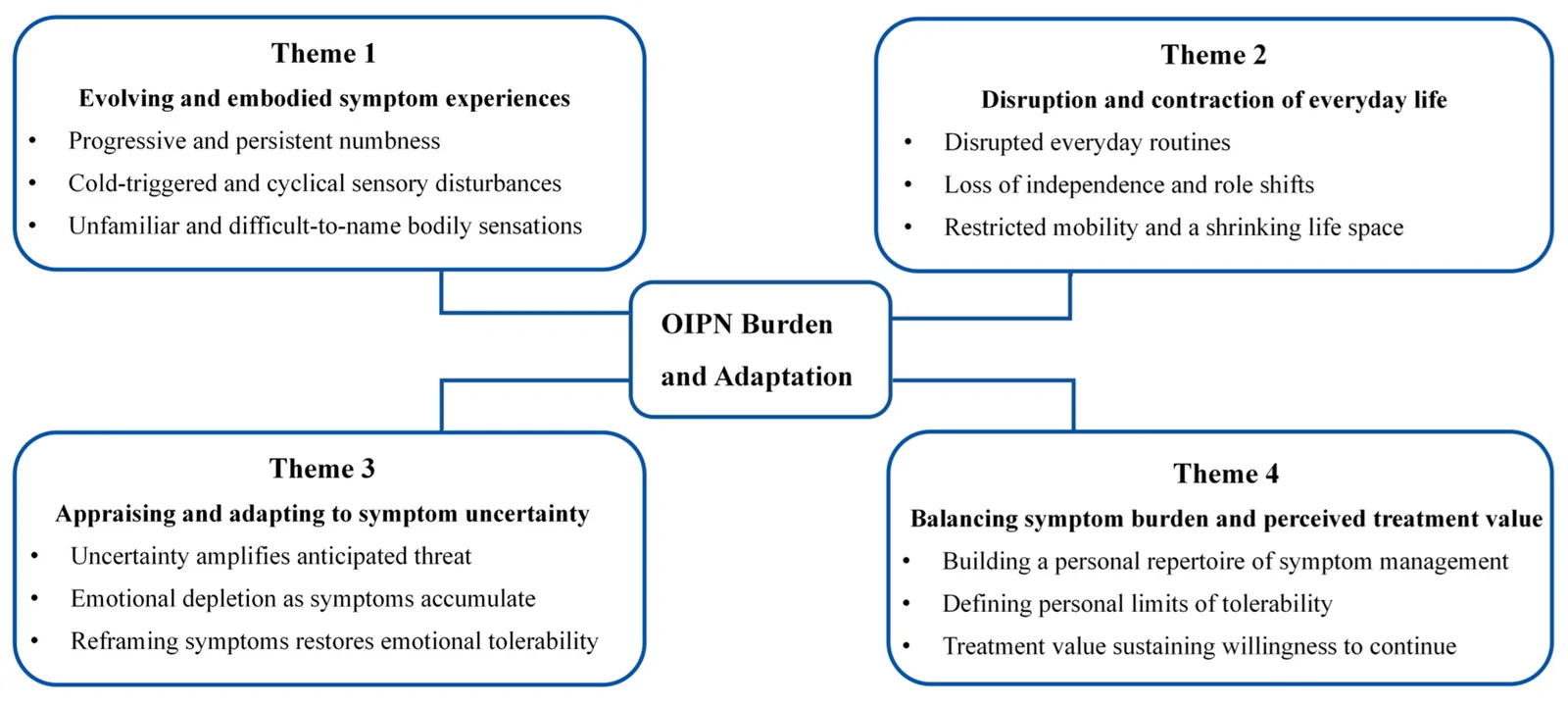
Themes and subthemes of OIPN symptom burden and adaptation.

We constructed four overarching themes, each comprising three subthemes, as shown in Figure 1.

#### 3.2.1. Theme 1: Evolving and Embodied Symptom Experiences

Participants retrospectively described both transient, recurrent disturbances and increasingly persistent, unfamiliar bodily experiences across chemotherapy cycles, making neuropathic symptoms more difficult to predict and ignore.

##### Progressive and Persistent Numbness

Numbness became more persistent across treatment cycles. Although it often eased between earlier cycles, participants described longer-lasting symptoms as chemotherapy continued.

*“My hands and feet are really numb, last time after chemo it got so bad. It’s been more than two weeks, and it’s still numb even now.” [P6]*

Persistent numbness was noticeable during routine movements or light touch, making the symptoms difficult to ignore in everyday activities.

*“Like now, if I just press my fingertips a bit, or even move them slightly, I can feel all five fingers are numb, the same with my feet.” [P8]*

The distribution and intensity of numbness varied, with some participants more affected in the feet and others in the hands or upper limbs.

*“I think my feet are better than my hands, from my fingers up to my arm it all goes numb. The hand with the injection of vein is worse, the other one is lighter.” [P10]*

##### Cold-Triggered and Cyclical Sensory Disturbances

Participants described cold-induced symptoms as abrupt, environment-triggered bodily reactions. These sensations could be accompanied by marked bodily discomfort, increasing both physical and emotional burden.

*“My hands feel like they’re being stabbed with needles. If I touch cold water, the pain is unbearable, even my hair stands on end.” [P14]*

These symptoms typically emerged shortly after chemotherapy, subsided within about a week, and recurred in subsequent cycles. One participant vividly described this recurring process as “like a marathon.”

*“When I touch cold things or cold water, it’s like metal stabbing into my palms and fingers. It usually starts right after chemo, then gets better within a week. But the whole process feels like a marathon. It’s really exhausting.” [P1]*

##### Unfamiliar and Difficult-to-Name Bodily Sensations

Some bodily changes extended beyond familiar numbness or pain and were difficult for participants to name or interpret within their existing understanding of illness and treatment.

*“It feels like bugs crawling on my face, and my feet feel like they haven’t been washed for half a month.” [P7]*

#### 3.2.2. Theme 2: Disruption and Contraction of Everyday Life

As neuropathic symptoms became embedded in everyday life, their effects expanded from disrupted routines to altered roles, restricted mobility, and reduced participation beyond the home.

##### Disrupted Everyday Routines

Disruption extended into everyday routines, as cold-related pain or abnormal sensations made ordinary activities such as washing, drinking, and personal care difficult or uncomfortable.

*“When I squeeze toothpaste, my hand hurts. I don’t even use facial cleanser anymore. When it sits in my palm, it feels cold, and the pain is unbearable.” [P5]*

*“I used to only drink cold water, never hot. Now if I drink cold water, my mouth hurts everywhere. I don’t even dare touch it anymore.” [P13]*

##### Loss of Independence and Role Shifts

Routine household tasks became more difficult, and some participants described a shift from caregivers to care recipients. Reduced contribution to household work was accompanied by a sense of “uselessness” among some participants, particularly several women.

*“I used to do all the housework myself. Now when I try to help, they tell me not to touch anything and just rest. Sitting there watching everyone else work makes me feel a bit useless.” [P12]*

*“My husband works really hard. When he comes home, I can cook rice in the rice cooker. Sometimes I prepare the ingredients and let him do the stir-frying, because I can’t touch metal things.” [P10]*

##### Restricted Mobility and a Shrinking Life Space

For some participants, lower-limb numbness and weakness reduced confidence in standing and walking, with greater instability and concern about falling when symptoms were severe.

*“I can’t even stand for a minute. If there’s no chair, I just sit on the ground. My feet are so numb, and I’m already weak. Last time I almost fell, luckily, I grabbed onto the wall.” [P4]*

Reduced confidence in mobility also changed exercise and leisure routines. Some participants lowered the intensity or adapted the type of activity, while others gradually stopped activities they no longer felt able to perform safely or comfortably.

*“I used to follow the whole exercise routine, but now I’m worried I might lose my balance. I don’t dare do the faster movements anymore, so I just do some simple, slower exercises at home.” [P14]*

*“When I dance, I can feel that numbness under my feet. My steps just aren’t as flexible. I gradually stopped going.” [P15]*

Some participants went out less often, particularly when cold exposure worsened symptoms, and daily life became increasingly centered at home.

*“In winter it’s too cold outside, I barely go out. Even during holidays, I don’t visit relatives anymore, I just stay at home by myself.” [P10]*

#### 3.2.3. Theme 3: Appraising and Adapting to Symptom Uncertainty

With symptoms remaining unpredictable, participants tried to understand what they meant and whether they would resolve. These appraisals were accompanied by different emotional responses and ways of adapting.

##### Uncertainty Amplifies Anticipated Threat

Uncertainty was prominent because the course and reversibility of neuropathic symptoms were difficult to predict, leading participants to question how long symptoms would persist and whether they would resolve after treatment.

*“After all the chemotherapy is done, no more medication, no more injections, will these symptoms actually get better?” [P13]*

Unfamiliar bodily sensations added to this uncertainty; when participants could not readily explain them, some interpreted the sensations as more threatening.

*“I felt like saliva was dripping, but when I wiped it, there was nothing. I even told my husband, ‘Am I having a stroke?’” [P10]*

For some participants, uncertainty about symptom progression raised concerns about ongoing chemotherapy, particularly after observing others change treatment because of intolerable symptoms and wondering whether they might face a similar situation.

*“I’m worried that if I continue chemotherapy, the symptoms might get worse. Someone in my ward had to switch medication because they couldn’t tolerate it anymore. If my symptoms become that severe, would I also need to change treatment?” [P15]*

A few participants also described anticipatory reactions before chemotherapy, sometimes beginning as soon as they arrived at the hospital.

*“After the numbness I had last time, I expected it to happen again. As soon as I arrived at the hospital, my hands already felt numb. I knew it might be psychological, but I still felt nervous and unsure what to do.” [P12]*

##### Emotional Depletion as Symptoms Accumulate

For some participants, recurrent numbness and pain, compounded by their impact on daily life, became exhausting. Distress ranged from fear to a sense of being unable to endure further treatment.

*“I’m already scared. Continuing chemotherapy feels like it might kill me. I’m really terrified. After the last session, I went home and felt so miserable (wipes tears), I really can’t take it anymore.” [P3]*

Others described frustration and irritability when repeated discomfort remained difficult to relieve.

*“By the fifth cycle, I was in a really bad mood. When my hands and feet feel warm, they itch; when they’re cold, they hurt, it’s unbearable. I get so frustrated that I stomp my feet hard, and that seems to help a little.” [P14]*

##### Reframing Symptoms Restores Emotional Tolerability

Not all participants viewed persistent neuropathy as equally threatening. Some came to see symptoms as a familiar, temporary, or unavoidable part of treatment, which appeared to lessen their emotional impact.

*“I have a good mindset. These symptoms are inevitable with oxaliplatin. It’s not just happening to me; others experience them too.” [P2]*

Some participants reframed symptoms as a difficult but purposeful journey with an attainable endpoint. This perspective appeared to sustain hope and make the recurring burden more emotionally tolerable.

*“It’s just like a marathon. As long as I keep going until the end, I believe I’ll make it.” [P1]*

For some participants, the importance attached to neuropathy depended on its effect on everyday life. When usual activities remained largely unaffected, symptoms were described as less important or less worthy of attention.

*“I don’t care much. As long as it doesn’t interfere with my normal life, I just don’t take it seriously.” [P9]*

Family members sometimes shifted attention from immediate discomfort toward signs of recovery or treatment progress, helping participants place neuropathy within a more hopeful account of their illness.

*“My son told me, ‘You’ve already come through when your condition was much worse. Now your complexion is better and you’ve gained weight, what are you afraid of?’ I also believe that once the drug effect wears off, I will recover naturally, and probably quite quickly.” [P1]*

#### 3.2.4. Theme 4: Balancing Symptom Burden and Perceived Treatment Value

Self-management, perceived tolerability, and the perceived value of treatment became closely linked in participants’ views about whether and how to continue chemotherapy.

##### Building a Personal Repertoire of Symptom Management

Participants developed individualized strategies to reduce discomfort and maintain daily functioning, particularly by avoiding cold-related stimuli such as cold water, metal objects, and low-temperature environments.

*“I avoid touching things like faucets, metal spoons, or ceramic bowls. I’ve switched to plastic bowls, and I’ve even wrapped door handles at home with cloth. When I go out, I always wear gloves.” [P5]*

Beyond cold avoidance, participants used warming, light physical activity, foot soaking, and medication to relieve persistent discomfort. Advice came from healthcare professionals, other patients, and online sources.

*“I walk around in the living room and rub my hands frequently. Moving more helps a bit, and keeping warm is essential. I’ve become almost like half a doctor myself. I look things up online whenever I have questions.” [P11]*

##### Defining Personal Limits of Tolerability

Tolerance of persistent symptoms varied. Some participants described themselves as accustomed to enduring physical discomfort and remained willing to continue treatment despite ongoing neuropathy.

*“I can tolerate it. I’m not someone who complains easily. I usually wait a long time before going to the hospital. I can endure it. It’s okay.” [P14]*

Others considered modifying treatment if symptoms worsened, while still viewing continuation of chemotherapy as necessary.

*“At first, I thought the numbness was something I could put up with because it would ease before the next treatment. But after the last two cycles, it never really went away. I told the doctor that if it kept getting worse, I would need a break or a longer interval before the next treatment. I still wanted to continue chemotherapy, but I felt I needed more time to recover.” [P3]*

For some, however, neuropathy reached a point at which it was no longer acceptable and continuing treatment came into question.

*“When the numbness became too severe, I told the doctor I didn’t want to continue. I just couldn’t tolerate it anymore.” [P6]*

##### Treatment Value Sustaining Willingness to Continue

For some participants, whether neuropathy remained acceptable depended partly on the perceived value of continuing anticancer treatment. When survival or disease control took priority, even worsening symptoms could still be viewed as a burden to endure.

*“For us, numbness in the hands and feet is a minor issue. Survival is what matters most. Even if it gets worse, treatment remains the priority.” [P4]*

*“As long as the treatment is still working, I think the numbness is something I can put up with. The cancer is the bigger concern for me. Even if my hands and feet get a little worse, I would still want to continue treatment.” [P7]*

## 4. Discussion

### 4.1. Main Findings

This study aimed to explore how patients experience, appraise, and adapt to OIPN during oxaliplatin treatment and how these experiences relate to considerations about treatment continuation. Previous qualitative studies have described the sensory, functional, and everyday impacts of OIPN, together with patients’ efforts to manage symptoms [12,13]. Our findings extend this evidence by highlighting how patients appraised and adapted to OIPN within the context of ongoing oxaliplatin treatment, and how symptom burden, perceived manageability, and perceived treatment value were reflected in treatment-related considerations. Together, these findings suggest that perceived tolerability was not determined by symptom severity alone.

Cold sensitivity, numbness, and other sensory disturbances are well-recognized features of OIPN and can disrupt everyday life. Prospective clinical evidence shows that neuropathic symptoms may intensify across treatment cycles and remain incompletely resolved between oxaliplatin administrations [20]. Participants’ retrospective accounts echoed this pattern but added the experience of living through it: recurrent symptoms, longer recovery, and incomplete resolution made the course of OIPN harder to anticipate, so uncertainty about when symptoms would subside became part of the burden itself. One participant’s description of OIPN as “like a marathon” captured this cycle of recovering from one episode while anticipating its return with the next treatment cycle.

Participants described restrictions in daily activities, independence, and participation beyond the home, supporting previous qualitative studies of OIPN [12,13]. A large survey of cancer survivors likewise identified household chores, social activities, hobbies, and walking among the activities most commonly affected by CIPN [21]. This contraction of everyday life may matter clinically because loneliness and limited social support are associated with poorer mental health and quality of life among cancer survivors [22,23]. In our data, functional limitations also affected participants’ sense of usefulness and contribution within the family. Similar disruptions of household activities and family responsibilities have been reported among women undergoing breast cancer treatment [24]. The pattern was particularly salient among several women in our study, suggesting that the meaning of OIPN may vary with valued roles and role expectations. Assessment should therefore extend beyond symptom severity and physical functioning to consider independence, social participation, and valued roles.

Participants often interpreted bodily changes against what they already knew from previous treatment cycles. Unfamiliar or unexpected sensations could unsettle that frame of reference; one participant initially feared that an unusual facial sensation might indicate a stroke. This pattern is consistent with research showing that patients receiving systemic anticancer therapy may struggle to judge whether symptoms are expected or warrant clinical attention [25] and with a recent qualitative synthesis describing symptom appraisal as iterative and informed by knowledge and prior treatment experience [26]. Repeated exposure also changed appraisal in different ways: recurrent symptoms could become more recognizable and less alarming, yet previous episodes could heighten vigilance before subsequent chemotherapy. A few participants even described numbness-like sensations before oxaliplatin administration. Within the Stress, Appraisal and Coping model, appraisal and coping change as demands and available resources are reassessed [14,15]. When coping resources were perceived as insufficient, anticipatory monitoring itself could become burdensome, leaving patients alert to possible worsening and uncertain consequences.

Persistent OIPN was not uniformly experienced as threatening. When symptoms became familiar or expected, or when daily functioning remained largely intact, some participants viewed neuropathy as a more acceptable part of treatment. This is consistent with evidence that symptom appraisal depends not only on the symptom itself but also on the meanings patients construct within their personal and treatment contexts [27]. Reframing was part of this adaptation. The “marathon” metaphor captured how an exhausting, recurrent burden could still be understood as a difficult but finite course with an endpoint. Appraisal also drew on information from others. Qualitative work indicates that patients’ information needs evolve across the cancer trajectory [28], while social context can influence symptom appraisal and help-seeking [29]. In our study, reassurance from family and recognition that others had similar symptoms were associated with less concern in some accounts; by contrast, participants who had seen peers change treatment because of neuropathy described greater threat. These accounts show that the meaning of symptoms varied with both personal experience and social information. Assessment should therefore explore how patients interpret and normalize symptoms, not only how severe those symptoms are. Individualized information and appropriate involvement of family or peers may support adaptation without normalizing clinically significant deterioration.

How manageable OIPN felt depended partly on the resources participants believed they had available. Given the limited availability of effective interventions, participants developed individualized self-management strategies to reduce discomfort and maintain everyday functioning, consistent with the range of patient-initiated approaches reported in previous CIPN studies [10,30]. Manageability was not simply a matter of individual capacity. Broader cancer self-management research shows that access to support may be constrained by limited awareness of available services and by problems with accessibility or usability [31,32], and previous studies have identified gaps in oncology professionals’ knowledge and provision of CIPN information and support [33,34]. When available strategies and support helped preserve functioning or restore a sense of control, persistent neuropathy could remain manageable. When they no longer did so, participants began to question whether their existing coping resources were enough.

Perceived symptom manageability, however, was not the sole basis on which participants considered treatment continuation. They also weighed the persistence and functional consequences of neuropathy against the perceived value of chemotherapy and what might be lost if treatment were modified or discontinued. Recent preference studies support this interpretation, showing that anticipated symptom permanence is associated with greater willingness to alter neurotoxic chemotherapy, while the availability of alternative treatments, perceived consequences of discontinuation, and expected survival benefit are important considerations in treatment preferences [35,36]. In participants’ accounts, willingness to continue chemotherapy despite worsening neuropathy was often expressed when disease control or survival was prioritized, whereas treatment delay, dose modification, or discontinuation was considered when symptoms were persistent, disruptive, or difficult to manage. Participants differed in the circumstances under which OIPN was regarded as unacceptable, with these judgments reflecting differences in symptom consequences, coping resources, expectations of recovery, and treatment priorities. Patients may therefore benefit from repeated opportunities throughout treatment to discuss whether neuropathy remains acceptable in relation to their treatment goals.

### 4.2. Implications for Practice and Research

Oncology nurses should assess OIPN across treatment cycles, considering not only symptom severity but also symptom persistence and recovery, effects on daily functioning, mobility and valued roles, and patients’ expectations regarding symptom reversibility. In patients with lower-limb numbness, weakness, gait instability, or balance problems, fall risk should also be assessed, with severe or worsening functional impairment prompting timely clinical evaluation and appropriate preventive measures. This multidimensional assessment may help identify unmet informational and self-management needs and guide individualized support. When neuropathy becomes persistent, functionally disruptive, or difficult to tolerate, nurses should explore how patients weigh symptom burden against the perceived value of chemotherapy and facilitate timely treatment-related discussions. Future longitudinal or mixed-method studies could combine qualitative interviews with validated patient-reported outcome measures to examine how OIPN symptoms, symptom appraisal, adaptive responses, and treatment-related considerations evolve across the course of oxaliplatin therapy.

### 4.3. Limitations

Several limitations should be considered. First, this single-center study included a relatively small sample, predominantly comprising patients with colorectal cancer, which may limit the transferability of the findings to other cancer populations and treatment regimens. Second, all participants were recruited in Shanghai, where oncology resources are relatively well developed; thus, the findings may not fully reflect the experiences of patients in resource-limited settings. In addition, patients who had already discontinued oxaliplatin were not represented, and experiences surrounding treatment modification or discontinuation may therefore be underrepresented. Finally, because participants were interviewed only once, changes across chemotherapy cycles were described retrospectively rather than observed longitudinally, and subsequent treatment decisions were not verified. Therefore, findings regarding treatment continuation reflect participants’ expressed considerations rather than confirmed decisions. Repeated interviews and more detailed treatment-context data may provide a fuller understanding of these issues.

## 5. Conclusions

This study shows that OIPN during oxaliplatin treatment was experienced as a recurrent and changing burden that could become increasingly persistent and extend into everyday life. Patients appraised and adapted to this burden within their broader personal and treatment contexts, with its meaning and perceived manageability varying in relation to functional consequences, expectations of recovery, and available coping resources. Considerations about continuing treatment reflected how symptom burden and tolerability were weighed against the perceived value of chemotherapy, rather than symptom severity alone. OIPN care should therefore extend beyond toxicity grading to consider patients’ appraisals, adaptation, symptom manageability, and treatment priorities during ongoing treatment.

## Figures and Tables

**Table 1:** Interview guide.

Interview Questions
1. Since starting oxaliplatin-based chemotherapy, can you tell me about the symptoms or changes you have experienced?
2. How have these symptoms affected your everyday life?
3. How have you made sense of these symptoms during treatment?
4. What have you done to deal with these symptoms?
5. Have these symptoms affected how you feel about your treatment?

**Table 2:** Participant characteristics (*N* = 15).

Participant	Age, y	Gender	Education	Cancer Site	Cancer Stage	Regimen (Cycle)	Cumulative Oxaliplatin Dose, mg	OIPN Grade
P1	65	Female	High school	Colon	IV	XELOX (2)	300	2
P2	62	Male	Junior high school	Colon	III	XELOX (5)	1000	1
P3	72	Female	Junior high school	Colon	III	XELOX (5)	880	2
P4	60	Male	Primary school	Rectal	IV	FOLFOX (9)	1150	3
P5	40	Female	High school	Gastric	II	SOX (5)	850	2
P6	68	Female	Junior high school	Colon	III	XELOX (6)	1200	3
P7	56	Female	Primary school	Gastric	IV	SOX (6)	1200	1
P8	72	Male	Primary school	Colon	III	XELOX (5)	1000	2
P9	69	Male	High school	Rectal	II	XELOX (8)	1600	1
P10	70	Female	Junior high school	Rectal	III	XELOX (6)	900	2
P11	68	Male	Junior high school	Gastric	III	SOX (6)	1150	1
P12	74	Female	Primary school	Rectal	III	XELOX (6)	1200	2
P13	60	Male	Primary school	Rectal	II	XELOX (5)	950	1
P14	65	Female	Junior high school	Gastric	III	XELOX (6)	900	3
P15	53	Female	University	Rectal	III	XELOX (7)	1210	2

Abbreviations: FOLFOX, folinic acid, fluorouracil, and oxaliplatin; OIPN, oxaliplatin-induced peripheral neuropathy; SOX, S-1 plus oxaliplatin; XELOX, capecitabine plus oxaliplatin.

## Data Availability

The data supporting the findings of this study are available from the corresponding author, Xiaoxia Qiu, upon reasonable request. The data are not publicly available to protect participant privacy.
